# Adsorption of ^137^Cs on titanate nanostructures

**DOI:** 10.1007/s10967-014-3228-5

**Published:** 2014-06-22

**Authors:** Barbara Filipowicz, Marek Pruszyński, Seweryn Krajewski, Aleksander Bilewicz

**Affiliations:** Centre of Radiochemistry and Nuclear Chemistry, Institute of Nuclear Chemistry and Technology, 03-195 Warsaw, Poland

**Keywords:** Adsorption, Nanotitanates, ^137^Cs, Titanate nanostructures

## Abstract

Various types of sodium and potassium titanate nanostructures (nanotubes, nanofibers, nanoribbons, nanwires) were synthesized and characterized by X-ray diffraction, SEM and TEM, as well BET and BJH methods. Adsorption of radiotracer ^137^Cs^+^ ions from aqueous solutions on synthesized titanate nanostructures was investigated in batch technique as a function of contact time, concentration of sodium ions and pH of the solutions. It was found that among the studied nanostructures nanotubes shows the highest selectivity for ^137^Cs, which is related to a zeolitic character of Cs^+^ adsorption. The efficient adsorption of ^137^Cs was obtained in Na^+^ solutions with concentration below 10^−2^ M, at pH 7–9 and in contact time above 2 h. Moreover, nanotubes have the higher specific surface area than other nanostructures, which results in better availability of ion exchange groups and high ion exchange capacity. These properties of nanotubes indicate that they may be used for adsorption of ^137^Cs from various types of nuclear wastes.

## Introduction

Radionuclides of ^134^Cs and ^137^Cs with the half-lives of 2 and 30 years, respectively, belong to the main long-lived fission products of ^235^U. These radionuclides undergo radioactive decays with the emission of beta particles and relatively strong gamma radiation. Cesium salts, the most common form of the element easily dissolved in water, causes a serious hazard if an accident appears with a nuclear reactor, such as that occurred, at Three Mile Island in Pennsylvania in 1979, Chernobyl in 1986 and the last big accident in 2011 at Fukushima, Japan, where thousands of cubic meters of sea water was used to cool reactor [1]. The cesium radionuclides that escaped into the environment are a serious threat to the health of present and future generations. Many years of observation after Chernobyl catastrophe indicates that leakage of ^137^Cs increases the risk of radioactive exposure of population because cesium isotopes could accumulate in mushrooms [2], lichens [3], mosses [4] and in higher plants such as grasses, ferns, heathers and blueberries [5] from which can be directly or indirectly transferred to other living organisms, including humans. When ^137^Cs enters into the organism, it allows the radioactive material to be dispersed throughout the body with higher concentration in muscle tissues. Therefore, serious attention has been paid to the removal and separation of ^137^Cs from radioactive waste solution in an economical and safe manner.

Various approaches and technologies such as co-precipitation with ferrocyanides [6] and ammonium molybdophosphates [7], solvent extraction with dicarbollylcobaltate(III) [8], macrocyclic ethers [9] and ion exchange have been developed for separation and immobilization of radioactive aqueous wastes generated at different stages of nuclear fuel cycles. In the case of ion exchangers many inorganic sorbents, such as ferrocyanides of transition metal cations [10], ammonium molybdophosphate [11], zeolites [12], clay minerals [13], titanium dioxides [14], sodium titanates [15] and zirconium phosphates [16] have been systematically studied for separation of ^137^Cs from nuclear waste water and safe disposal of the exchanged cations. The advantage of this approach is the ability of these materials to withstand intense radiation and elevated temperatures in addition to their high selectivity for Cs^+^ ions.

Nanostructures of titanates play an important role in the process of binding inorganic cations, including radionuclides, because of their good sorption properties. The advantage of some layered nanotitanates is the collapse of their structure, which occurs during the ion exchange and results in tight immobilization of targeted cations in the interlayer, thus in irreversible ion exchange [17–21].

The aim of the present work was to evaluate and compare selectivity of various titanate nanostructures for radionuclide of ^137^Cs from aqueous solutions.

## Experimental

### Materials

All chemicals were analytical pure grade and used without further purification. The solutions were prepared in deionized water with electrical conductivity lower than 10 μS cm^−1^ at 25 °C (Millipore, Direct-Q3). Crystalline anatase and amorphous grains of TiO_2_ were purchased from Sigma Aldrich. Radionuclide ^137^Cs in the form of ^137^CsCl was supplied by the Radioisotope Centre POLATOM, National Centre for Nuclear Research (Świerk, Poland). All pH measurements were made by CP-501, ELMETRON pH meter.

### Synthesis of titanate nanostructures

Various titanate nanostructures were prepared via hydrothermal process according to procedures described by Kasuga et al. [22]. As precursors crystalline anatase and amorphous forms of TiO_2_ were used. Briefly, 1.5–1.7 g of the precursor was mixed with 70 mL of 10 M NaOH or KOH and the suspension was placed into a PTFE (polytetrafluoroethylene—Teflon) lined-autoclave heated at 140 or 200 °C for 24–72 h with constant stirring. After cooling to room temperature the obtained product was filtered, rinsed with water, next with 0.1 M HCl and again several times with water until the pH of the supernatant solution reached a constant value of ca. pH 8–9. In the case of nanowires prepared with KOH, the final precipitate was first rinsed with NaOH to transform them into Na-form followed by acid–water washing as described above. Finally, the Na-titanate nanostructures were dried at 70 °C for 8 h, Table 1.

**Table 1:** Conditions used to prepare various titanate nanostructures

	Precursor material	Alkaline solution	Temperature (°C)	Reaction time (h)	Ref.
Nanotubes	Anatase	10 M NaOH	140	72	[18–20, 23]
Nanowires	Anatase	10 M KOH	140	24	[23]
Nanofibers	Amorphous grains	10 M NaOH	140	24	[18, 23]
Nanoribbons	Amorphous grains	10 M NaOH	200	24	[23]

### Characterization of the nanostructures

Titanate nanostructures were characterized by X-ray diffraction analysis (XRD) using the Bruker D8 ADVANCE diffractometer equipped with Cu Kα radiation source. The specific surface area was measured by the Brunauer–Emmett–Teller isotherm (BET) method and the pore size distribution by the Barrett–Joyner–Halenda (BJH) method using classical adsorption/desorption nitrogen isotherms on a Micromeritics ASAP 2405 instrument. The morphology of the titanate nanostructures was analyzed by the scanning electron microscope (SEM) Zeiss ULTRA plus and transmission electron microscope (TEM) LIBRA PLUS 120 EF.

### Radioactivity measurements

Gamma-spectrometry was carried out using a calibrated intrinsic Ge detector (crystal active volume 100 cm^3^) and PC-based Multichannel Analyzer (MCA, Canberra). The detector had a resolution of 0.8 at 5.9 keV, 1.0 at 123 keV, and 1.9 at 1,332 keV. Samples were measured after 30 min of phase separation when the secular equilibrium of ^137^Cs with its decay product of ^137m^Ba (*T*_1/2_ = 2.55 min) was achieved. The 662 keV gamma-line was used.

### Kinetics of cation exchange

The kinetics of ^137^Cs^+^ cations adsorption on nanostructures were studied by shaking 30 mg of the adequate sorbent with 20 mL of the 0.1 M NaNO_3_ solution containing ^137^Cs radiotracer. At designated time intervals from 2 min to 24 h, a 1 mL of the suspension sample was collected, centrifuged and an aliquot of 0.5 mL of the supernatant was taken for the radioactivity measurement.

### Measurements of distribution coefficients

The distribution coefficients of ^137^Cs cations on various titanate nanostructures were determined by batch technique. The mass of titanate sample was 10 mg and total volume of solution was 4.5 mL. The *K*_d_ values were calculated according to the equation:

$$ K_{\text{d}} \; = \;\frac{{ (A_{\text{i}} - A_{\text{eq}} )}}{{A_{\text{eq}} }}\frac{V}{m}, $$

where *A*_i_ and *A*_eq_ denote the radioactivity of the initial solution and at the equilibrium, respectively, *V* is the volume (mL) of the solution, and *m* (g) is the mass of the titanate adsorbent [24].

### Determination of ion exchange capacity (IEC)

The total IECs of titanate nanoparticles for Cs^+^ ions were determined by batch equilibration of 0.5 g of solid material with 4.5 mL of 0.1 M CsCl solutions spiked with ^137^Cs. Initial activity (*A*_i_) and activity after attaining of equilibrium (*A*_eq_) were measured and IEC was calculated according to the equation:

$$ {\text{IEC}} = \frac{{ ( 1_{\text{i}} - A_{\text{eq}} )}}{{A_{\text{i}} }}\frac{CV}{m}, $$

where *C* is the concentration of CsCl, and *V* volume of the solution, and *m* (g) is the mass of the nanotitanate adsorbent.

## Results and discussion

### Identification and characterization of nanostructures

**Fig. 1:**
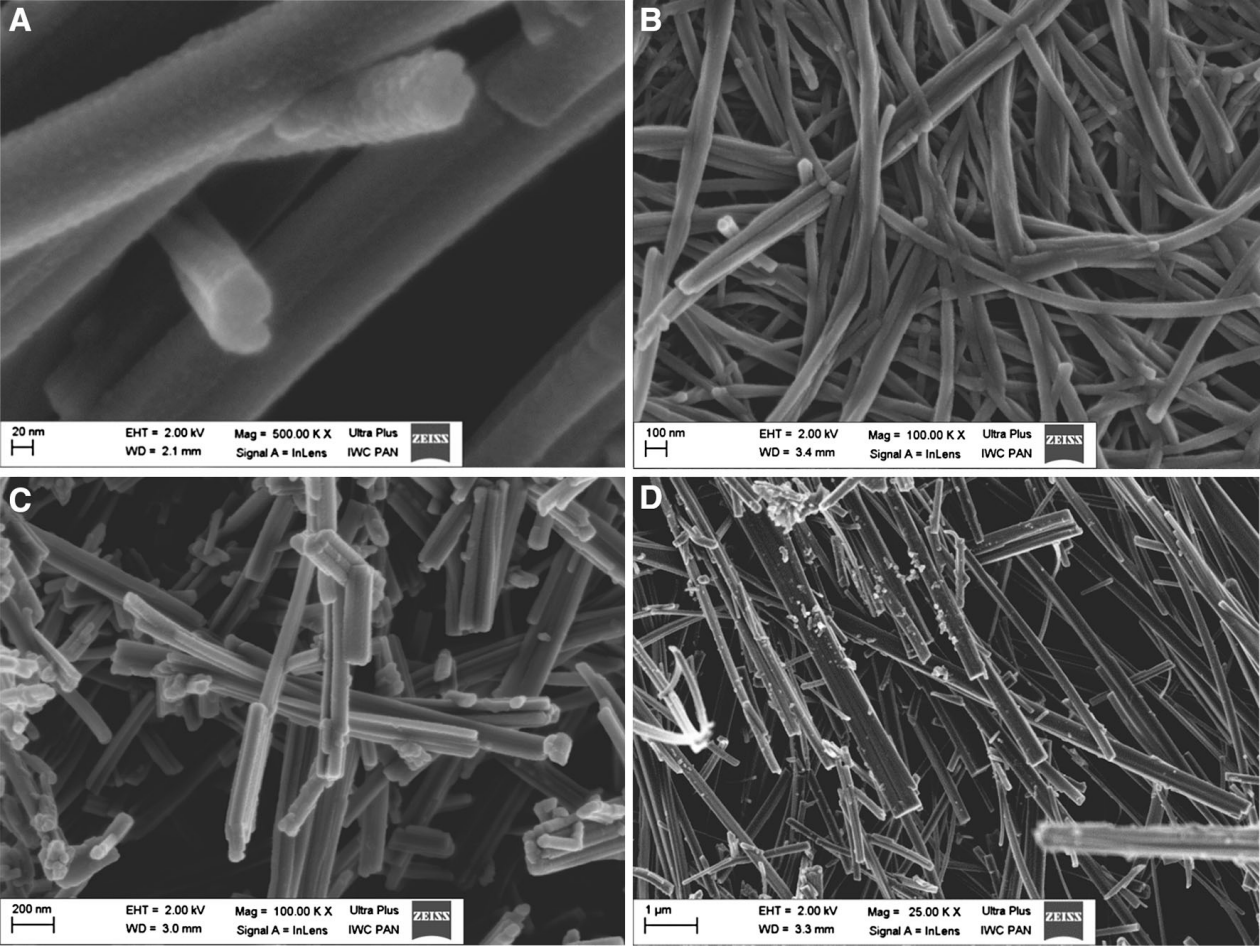
SEM images of **a** nanotubes, **b** nanowires, **c** nanofibers, **d** nanoribbons

The morphology of the four synthesized nanostructures is shown in Fig. 1. The solid displays an aggregated shape with heterogeneous morphological distribution of diverse polyhedral forms and particles diameters in the range of 10–100 nm and length of the microns order.

**Fig. 2:**
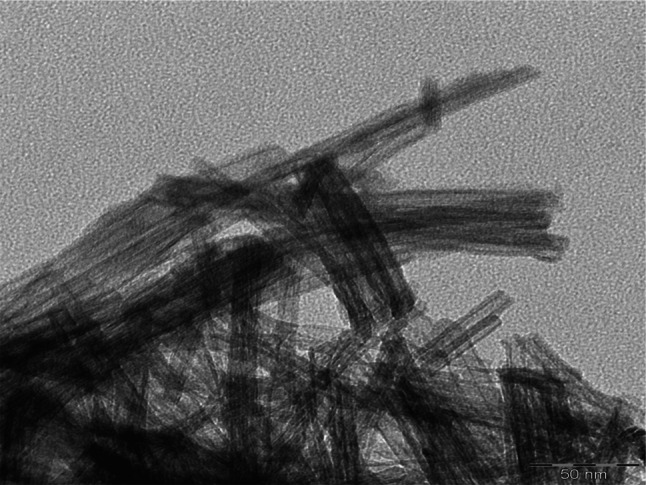
TEM image of nanotubes

Formation mechanism of titanate nanostructures is not clear and unexplained. The hydrothermal reaction of anatase with concentrated NaOH solution at the temperature of 140 °C resulted in the production of nanotubes (Fig. 1a) [21, 22]. Confirmation of the structure is also visible in the TEM image (Fig. 2). However amorphous TiO_2_ treated at the same temperature resulted in the production of nanofibers (Fig. 1b) [20, 23]. Amorphous TiO_2_ was used as a raw material to produce nanoribbons (Fig. 1c) [23] when the synthesis was performed with NaOH solution at temperature equal 200 °C. Whereas nanowires (Fig. 1d) were obtained in KOH [22, 23]. The crystalline structure of the crystalline TiO_2_ polymorphs such as anatase and rutile [22, 23] is described with representative TiO_6_ octahedra, which share vertices edges to build up the three-dimensional framework of oxides [19, 20]. It can be proposed that some of Ti–O–Ti bonds of the raw materials are broken when reacted with alkaline solution, and layered titanates composed of octahedral TiO_6_ units with alkali metal ions are formed in the form of thin small sheets [19–23]. Under autoclaving, the titanate sheets were exfoliated into nanosheets with one or two layers, and the nanosheets rolled into nanotubes with a slow growth rate possibly due to the high concentration of NaOH solution [18–20].

Simultaneously, nanosheets with several nanotrititanate layers were formed three-dimensionally, which may be difficult to roll up completely, so the edges of nanosheets were often bent. The Na^+^ ions attached in nanotubes and nanosheets could be exchanged and removed after washing with water and diluted by acid solution [19–20]. Titanium in amorphous TiO_2_ gel is octahedrally coordinated by both oxygen atoms, which are linked with titanium atoms of adjacent octahedra, and –O–H groups, possessing a different structure from crystalline titanium dioxide polymorphs. When reacted with NaOH, some of Ti–O–H (and Ti–O–Ti) bonds are broken by the cauterization of caustic soda solution to generate new coordinations [18–21]. Then, the octahedra interact between each other to give long-rang order, partly transform into layered structure of titanate, and self-assemble into thin titanate nanosheets. With the process of hydrothermal reaction, more and more nanosheets could be formed with anisotropic growth [17–20]. Some of individual sheets may merge to form nanofibers [20] and further interlink into a hierarchical intertextural structure [21, 23]. Most of nanofibers can interlink chemically rather than physically [20].

With the increase of the temperature (>160 °C), the nanoribbons grow accompanied with the decrease of titanate nanotubes [23]. And the crystalline structure transforms from trititanate to form more stable phase of pentatitanate H_2_Ti_5_O_11_·H_2_O [22]. Since the nanoribbons curve along the ribbon axis were observed, it is reasonable to believe that the morphology is temperature depended. The form of flat nanoribbons is stable during elevated temperature autoclaving. K_2_Ti_8_O_17_ nanowires were formed in the autoclaving of TiO_2_ powder and KOH solution [21, 23].

The specific surface area and pore size distribution of titanate nanostructures were characterized by the BET and BJH methods. The results are presented in Table 2.

**Table 2:** Specific surface area and pore size distribution of titanate nanostructures

Nanostructure	Specific surface area (m^2^ g^−1^)	Pore size distribution (m^3^ g^−1^)
Nanotubes	298.35	0.48
Nanowires	106.84	0.38
Nanoribbons	122.99	0.35
Nanofibers	127.05	0.37

As shown in Table 2 the all synthesized nanostructures have a well-developed surfaces, especially nanotubes, where specific surface area was three times greater than for the others. This should result in better availability of surface hydroxyl groups in nanotubes samples.

**Fig. 3:**
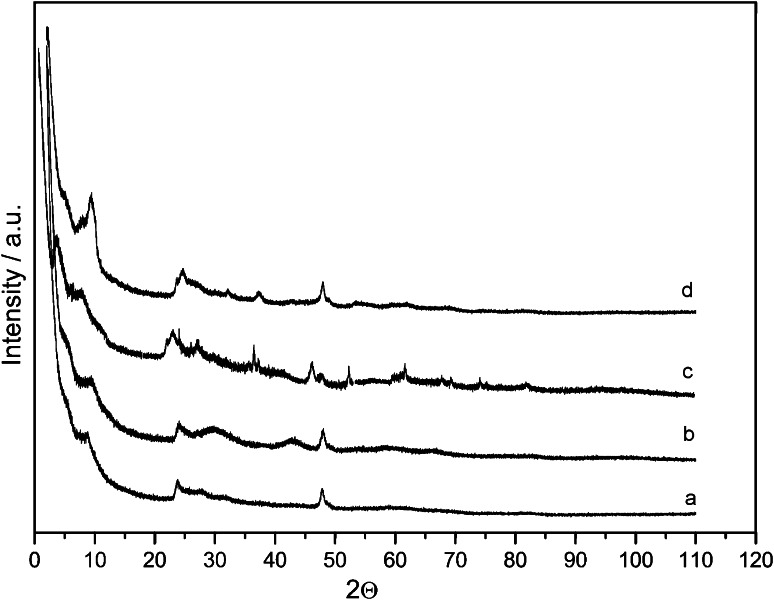
XRD pattern of *a* nanotubes, *b* nanowires, *c* nanoribbons, *d* nanofibers

Figure 3 shows XRD patterns of the obtained samples. The nanotitanate structures were identified by comparing relative intensities in their XRD with those reported in the literature [18–20, 23]. The XRD analysis confirmed the synthesis of various forms such as obtained in the work of Yuan et al. The four powder diffraction patterns were found to be in good agreement. The broadened XRD pattern peaks observed in the obtained samples are indicative of small size of the crystalline products.

### Adsorption properties of synthesized nanostructures

**Fig. 4:**
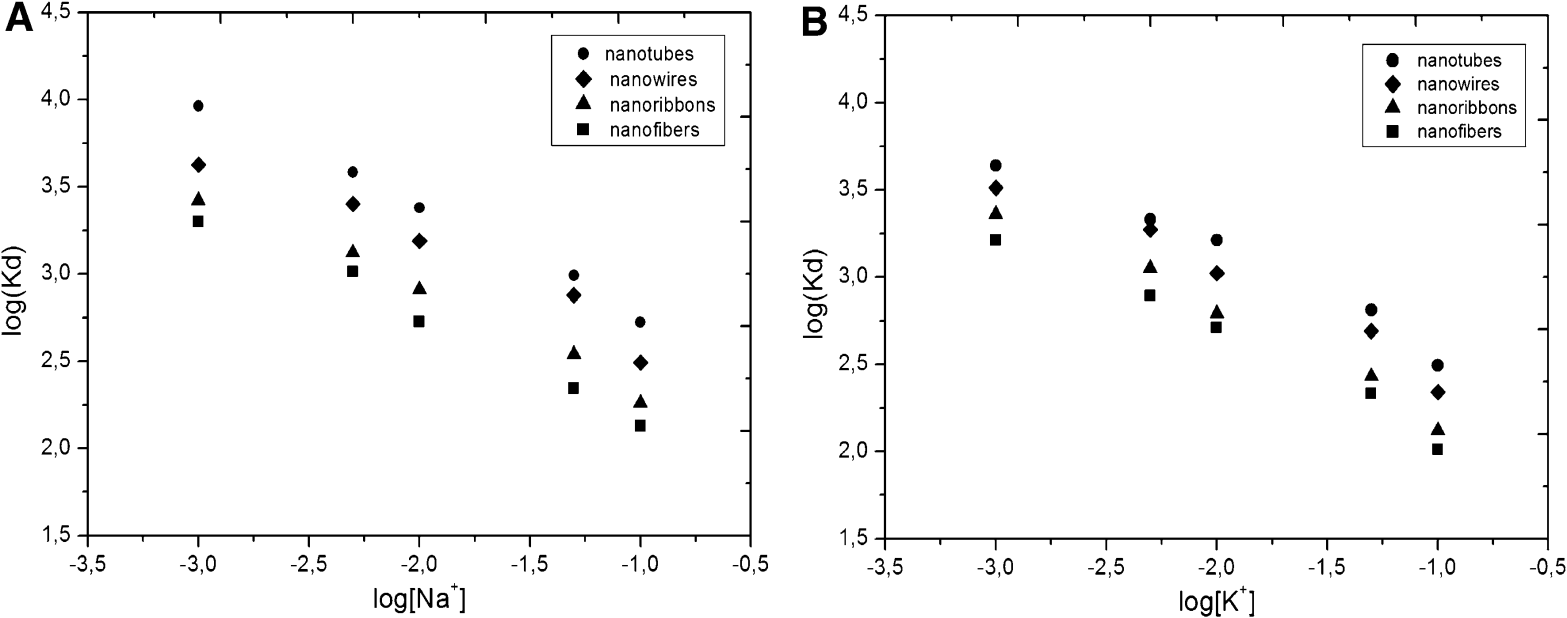
The dependence of ^137^Cs distribution coefficient on Na^+^ and K^+^

The selectivity of the obtained nanostructures was examined by adsorption studies of ^137^Cs from solutions containing different concentrations of NaNO_3_ or KNO_3_ (10^−3^ to 0.1 M). The dependence of ^137^Cs distribution coefficient on Na^+^ and K^+^ concentrations are shown in Fig. 3. As expected, a linear dependence of log*K*_d_ on log[Na^+^] and log[K^+^] confirms ion exchange mechanism of sorption. The *K*_d_ values for ^137^Cs^+^ ions decreased with increasing the NaNO_3_ or KNO_3_ concentrations due to the competition for ion exchange sites on the nanostructures surface. Since K^+^ cations have larger ionic radius than Na^+^ their influence on Cs^+^ adsorption was greater. As is presented in Fig. 4 titanate nanotubes had the highest values of *K*_d_ towards Cs^+^ ions. Probably, similarly as in the case of ferrocyanides and zeolites, Cs^+^ sorption can occur inside the nanotubes, where cations are dehydrated and the selectivity of adsorption depends on the energy of hydration. The inner sorption of Cs^+^ is relatively easy, since the energy of hydration for the Cs^+^ is rather small.

Effect of the specific surface area was also observed when examining the (IEC) of nanostructures. As shown in Table 3 the greater amount of the available ion exchange centers on nanotube sorbents caused their higher IEC.

**Table 3:** Ion exchange capacity (IEC) of synthesized nanostructures

Nanostructure	IEC (mmol g^−1^)
Nanotubes	1.17
Nanowires	0.94
Nanoribbons	1.02
Nanofibers	0.79

**Fig. 5:**
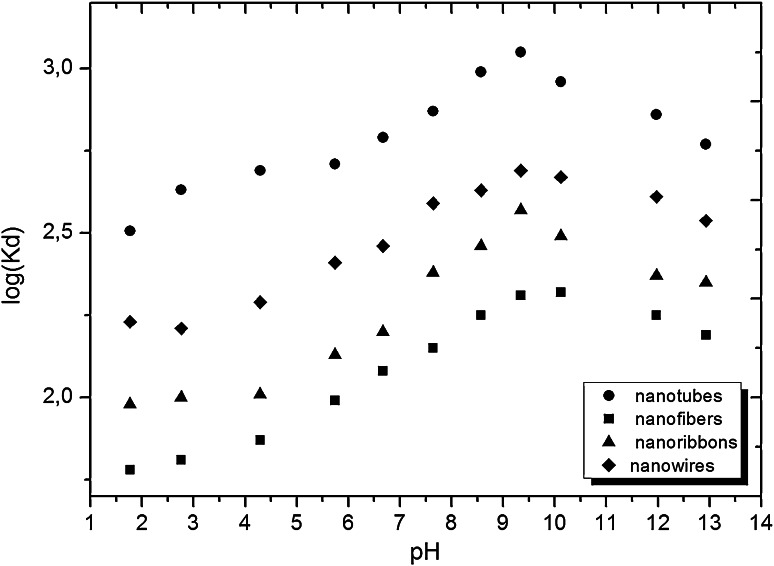
Effect of pH on the ^137^Cs adsorption on titanate nanostructures. Solution of phosphate buffer (0.1 M) was acidified by 1 M HNO_3_ or alkalized by 1 M NaOH

Since the hydroxyl functional groups of titanate sorbents are weakly acidic, the effect of pH on ^137^Cs adsorption was studied. The results are shown in Fig. 5.

As expected, increasing of *K*_d_ with increasing of pH was observed in pH range 2–9, which is related to a slightly acidic character of the hydroxyl functional groups. The observed results *K*_d_ values in pH above 9 seems rather unusual for sorption of inorganic cations on oxide sorbents, where the increase in pH is usually followed by a simultaneous increase in the *K*_d_ values. In the case of nanostructures the highest sorption of ^137^Cs was reached at pH 7–9, and at pH higher than 9 the *K*_d_ slightly decreased.

**Fig. 6:**
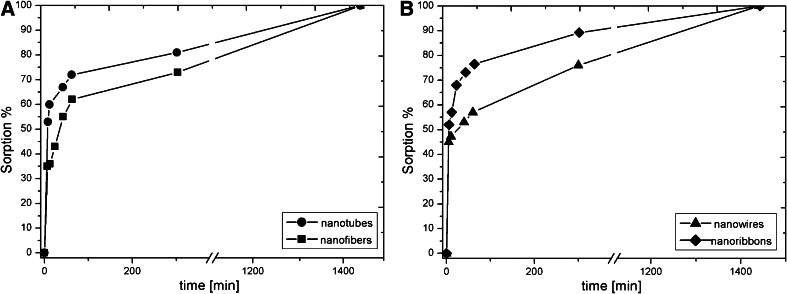
Sorption percentages of ^137^Cs on **a** nanotubes, nanofibers; **b** nanoribbons, nanowires as a function of time

Kinetics of ion exchange is one of the most important characteristics in defining the efficiency of the sorbent. Na-titanate nanostructures revealed high and fast initial sorption of ^137^Cs^+^, followed by apparent saturation, that was especially visible in the case of nanowires and nanotubes (Fig. 6). This can be explained by the fast initial sorption on the surface of the nanostructure, and a slower ion exchange inside the nanopores.

## Conclusions

The results presented show that nanotitanates, in particular nanotubes, have efficiently high selectivity for ^137^Cs. This is probably caused by a zeolitic character of Cs^+^ sorption where sorption of dehydrated cations occurs inside the nanotubes. Unfortunately, due to porous structure with small interlayer distance ion exchange kinetics are relatively slow, although 60 % of equilibrium was reached within 1 h. These ion exchange properties of nanotubes show that they may be useful for decontamination of non-acidic nuclear wastes and for long term disposal.
